# Quantitative In Vivo Imaging of ATP Dynamics in *Drosophila* Using the Ratiometric Biosensor QUEEN

**DOI:** 10.1111/dgd.70065

**Published:** 2026-08-03

**Authors:** Yuhan Luo, Sayaka Sekine, Naoto Kasahara, Hiroyuki Uechi, Takafumi Toyohara, Takaaki Abe, Erina Kuranaga

**Affiliations:** ^1^ Graduate School of Life Sciences Tohoku University Sendai Japan; ^2^ Department of Life Science and Technology Institute of Science Tokyo Tokyo Japan; ^3^ Laboratory for Histogenetic Dynamics, Graduate School of Pharmaceutical Sciences Kyoto University Kyoto Japan; ^4^ Tohoku University Graduate School of Biomedical Engineering Sendai Japan

**Keywords:** ATP biosensor, *Drosophila melanogaster*, energy metabolism, in vivo imaging, mitochondrial function, QUEEN, ratiometric sensor

## Abstract

Genetically encoded ATP biosensors enable monitoring of cellular energy status, but their application in multicellular organisms remains limited. QUEEN is a ratiometric ATP biosensor consisting of a bacterial ATP‐binding protein fused to a circularly permuted fluorescent protein and has been primarily validated in cultured cells. Here, we generated transgenic *Drosophila melanogaster* lines expressing QUEEN‐7μ, enabling tissue‐specific expression through the GAL4/UAS system. We characterized its performance in motor neurons and muscles. QUEEN‐7μ responses were validated using pharmacological and genetic perturbations of mitochondrial function. Mitochondrial inhibition decreased the QUEEN‐7μ ratio, consistent with reduced ATP levels, whereas acute treatment with mitochonic acid‐5 (MA‐5), a small molecule that enhances mitochondrial ATP synthesis, increased the QUEEN‐7μ ratio, with a larger effect at higher concentration. Consistently, genetic manipulations, including the mitochondrial Complex I knockdown and Mitofilin/MIC60 overexpression, produced corresponding decreases and increases in the QUEEN‐7μ ratio. These results establish QUEEN‐7μ as a reliable ratiometric ATP reporter for quantitative in vivo analysis in *Drosophila*. This system provides a versatile platform for investigating energy metabolism and mitochondrial function in *Drosophila*.

## 1 Introduction

Intracellular adenosine triphosphate (ATP) levels reflect the energetic state of cells and are essential for diverse physiological processes, including development, neuronal activity, and stress responses (Bonora et al. 2012). Quantitative measurement of ATP dynamics in living tissues is therefore critical for understanding how cellular energy metabolism is regulated in multicellular organisms.

Intracellular ATP concentration is generally maintained within the millimolar range by homeostatic metabolic regulation. A compilation of published cellular nucleotide measurements reports an average mammalian ATP concentration of 3.2 ± 1.7 mM (Traut 1994), and live‐cell ATP measurements using genetically encoded sensors have also confirmed millimolar ATP concentrations (Imamura et al. 2009). Although such buffering supports cellular function under fluctuating energy demands, mitochondrial perturbations can produce measurable changes in ATP levels, motivating direct visualization of ATP dynamics in living tissue.

Genetically encoded fluorescent ATP sensors have enabled monitoring of intracellular ATP levels in living systems (Rajendran et al. 2016). Among them, Forster resonance energy transfer (FRET)‐based sensors such as ATeam (Imamura et al. 2009) have been widely used in cultured cells and have also been applied in selected tissues and model organisms (Tsuyama et al. 2013). However, their application in vivo remains relatively limited compared to cell‐based applications, and quantitative comparisons across cells and tissues can be influenced by variations in expression levels and imaging conditions.

The ratiometric ATP biosensor QUEEN (QUantitative Evaluator of cellular ENergy) provides an alternative approach (Yaginuma et al. 2014). QUEEN consists of the ATP‐binding ε subunit of bacterial ATP synthase fused to a circularly permuted fluorescent protein and reports ATP levels as an excitation ratio. The ratiometric design enables quantitative measurement that is largely independent of sensor expression level and has been validated in microorganisms and cultured cells (Yaginuma et al. 2014; Takaine et al. 2019).

Despite these advantages, the application of QUEEN has been largely restricted to unicellular systems and in vitro cultured cells, and its performance in complex tissues of multicellular organisms remains unclear. Establishing an in vivo system for QUEEN would enable spatially and genetically controlled analysis of cellular energy states.

*Drosophila melanogaster* is a powerful model for developing genetically encoded biosensors due to its versatile GAL4/UAS expression system (Brand and Perrimon 1993) and extensive genetic toolkit (Wangler et al. 2015). An ATP reporter compatible with this system would facilitate tissue‐specific analysis of energy metabolism in vivo.

Here, we generated transgenic *Drosophila* strains expressing the ratiometric ATP biosensor QUEEN‐7μ under UAS (upstream activating sequence) control. Using tissue‐specific GAL4 drivers, we evaluated QUEEN‐7μ in motor neurons and skeletal muscles. Through pharmacological and genetic perturbations of mitochondrial function, we show that QUEEN‐7μ reliably detects bidirectional changes in ATP‐associated signals in *Drosophila* tissues. These findings establish QUEEN reporter flies as a robust tool for quantitative in vivo analysis of ATP dynamics in a genetically tractable multicellular organism.

## 2 Materials and Methods

### 2.1 Fly Stocks and Husbandry

All *Drosophila melanogaster* stocks were maintained on standard cornmeal‐yeast food at 25°C under a 12 h light/12 h dark cycle. The following fly lines were used: *D42‐GAL4* (BDSC #8816), *Mef2‐GAL4* (BDSC #27390), *UAS‐ND‐75 RNAi* (BDSC #27739), *UAS‐Mitofilin* (gift from Dr. Xinnan Wang, Stanford University; Tsai et al. 2017), *UAS‐QUEEN‐7μ* (this study; see below), and *w* ^*1118*^.

### 2.2 Generation of UAS‐QUEEN‐7μ Transgenic Flies

The pUASTattB‐QUEEN‐7μ plasmid was constructed as follows: A DNA fragment was amplified using KOD Plus Neo (TOYOBO, Japan) with pN1‐QUEEN‐7μ (Addgene, #129307) as the template and the following primer pair: K#1779 (“ggccgcggctcgagggtaccaacttaaaaaaaaaaatcaaaatggtgaaaacgatccacgtgagc”) and K#1780 (“aacgattcattctagtcacttcatttccgcaacgctcaaacg”). The pJFRC81‐10xUAS‐IVS‐Syn21‐GFP‐p10 plasmid (Addgene, #36432) was digested with KpnI and XbaI, and the amplified fragment was inserted using the In‐Fusion HD Cloning Kit (Clontech). The final construct was confirmed by sequencing. Transgenic flies at the attP40 site were generated by *Drosophila* Microinjection Services (WellGenetics Inc., Taiwan).

### 2.3 CCCP Treatment

**FIGURE 1:**
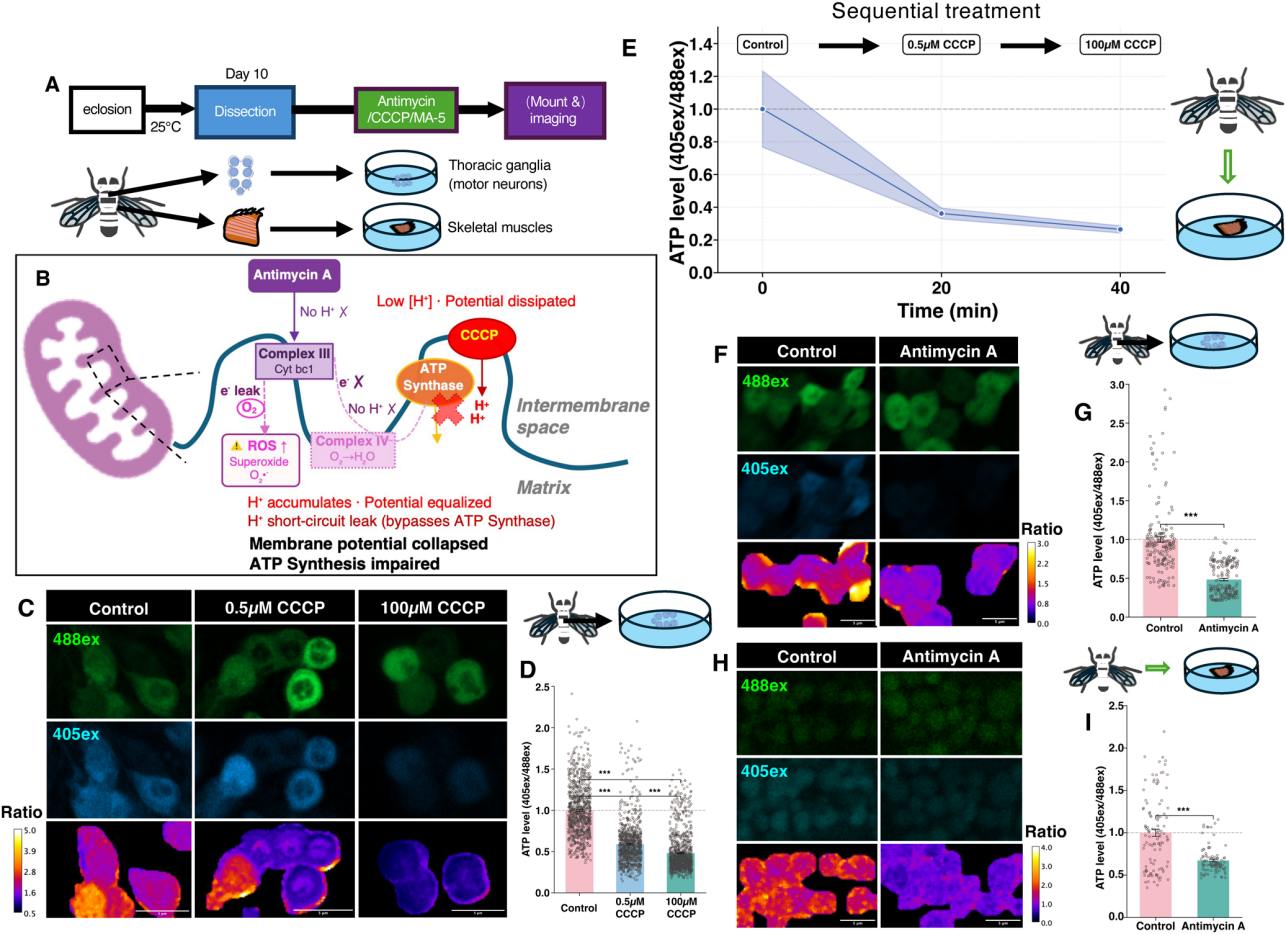
QUEEN‐7μ detects ATP reduction induced by mitochondrial inhibition in *Drosophila* tissues. (A) Schematic of the experimental workflow. Adult flies were aged for 10 days at 25°C following eclosion, dissected to prepare thoracic ganglia or skeletal muscle samples, treated with Antimycin A, CCCP, or vehicle for the indicated durations, mounted, and imaged by confocal microscopy. (B) Schematic of mitochondrial inhibition by Antimycin A and CCCP. Antimycin A inhibits Complex III, blocking electron transfer and proton pumping. CCCP acts as a protonophore that dissipates the mitochondrial membrane potential. Both treatments reduce the proton motive force required for ATP synthase. (C) Representative confocal images of thoracic ganglia from adult flies expressing QUEEN‐7μ in motor neurons treated with HL3.1 buffer alone (control), 0.5 μM CCCP, or 100 μM CCCP for 15 min. Images show 488ex excitation (top), 405ex excitation (middle), and pseudo‐colored 405ex/488ex ratio images (bottom, Fire LUT). (D) Quantification of QUEEN‐7μ ratio in motor neurons, expressed as the 405ex/488ex fluorescence ratio normalized to the control group mean. Each data point represents an individual cell. Data are presented as mean ± SEM. CCCP significantly decreased the QUEEN‐7μ ratio, with significant differences among all three groups (Kruskal–Wallis test followed by pairwise Mann–Whitney *U* tests with Bonferroni correction; ***p < 0.001). *N* indicates the number of animals and *n* indicates the number of cells analyzed per group (Control: *N* = 5, *n* = 875; 0.5 μM CCCP: *N* = 7, *n* = 904; 100 μM CCCP: *N* = 6, *n* = 1005). (E) Sequential exposure experiment in adult skeletal muscle expressing QUEEN‐7μ. Muscle preparations were exposed to vehicle (0 min, control), 0.5 μM CCCP (20 min), and 100 μM CCCP (40 min) by buffer exchange and imaged at the indicated time point. The ratio is expressed as the 405ex/488ex fluorescence ratio normalized to the control mean. Data were pooled from three independent experiments and are presented as mean ± SEM (shaded region). *N* indicates the number of preparations imaged and *n* indicates the number of AOIs analyzed per time point across all experiments (control: *N* = 11, *n* = 32; 0.5 μM CCCP: *N* = 13, *n* = 36; 100 μM CCCP: *N* = 18, *n* = 51). (F) Representative confocal images of thoracic ganglia from adult flies expressing QUEEN‐7μ in motor neurons treated with vehicle (control) or 25 μM Antimycin A. Images show 488ex excitation (top), 405ex excitation (middle), and pseudo‐colored 405ex/488ex ratio (bottom, Fire LUT). (G) Quantification of the QUEEN‐7μ ratio in motor neurons following 25 μM Antimycin A treatment, expressed as the background‐subtracted 405ex/488ex fluorescence ratio normalized to the control mean within each experiment. Each data point represents an individual cell. Data were pooled from three independent experiments and are presented as mean ± SEM. Antimycin A significantly decreased the QUEEN‐7μ ratio compared with control (Mann–Whitney *U* test, two‐sided; ***p < 0.001). *N* indicates the number of animals and n indicates the number of cells analyzed per group (control: *N* = 9, *n* = 180; 25 μM Antimycin A: *N* = 9, *n* = 180). (H) Representative confocal images of adult skeletal muscle from flies expressing QUEEN‐7μ in skeletal muscle treated with vehicle (control) or 100 μM Antimycin A. Images show 488ex excitation (top), 405ex excitation (middle), and pseudo‐colored 405ex/488ex ratio images (bottom, Fire LUT). (I) Quantification of the QUEEN‐7μ ratio in adult skeletal muscle following 100 μM Antimycin A treatment, expressed as the 405ex/488ex fluorescence ratio normalized to the control group mean. Each data point represents an individual AOI. Data are presented as mean ± SEM. Antimycin A significantly decreased the QUEEN‐7μ ratio, compared with control (Mann–Whitney *U* test, two‐sided; ***p < 0.001). *N* indicates the number of animals and *n* indicates the number of AOIs analyzed per group (control: *N* = 6, *n* = 109; 100 μM Antimycin A: N = 7, *n* = 98). Genotype: (C, D, F, G) *UAS‐QUEEN‐7μ/+; D42‐GAL4/+*; (E, H, I) *UAS‐QUEEN‐7μ/+; Mef2‐GAL4/+*.

For motor neuron experiments (Figure 1C,D), adult *Drosophila* aged 10 days post eclosion of the genotype *UAS‐QUEEN‐7μ/+; D42‐GAL4/+* were dissected in HL3.1 buffer prepared as previously described (Feng et al. 2004; Oka et al. 2021). Thoracic ganglia were incubated in HL3.1 buffer supplemented with either 0.5 or 100 μM CCCP (carbonyl cyanide 3‐chlorophenylhydrazone, Sigma‐Aldrich) for 15 min (Figure 1A). Following treatment, preparations were washed three times with HL3.1 buffer, mounted, and imaged on a Zeiss LSM980 confocal microscope.

For skeletal muscle ex vivo experiments (Figure 1E), adult *Drosophila* aged 10 days post eclosion of the genotype *UAS‐QUEEN‐7μ/+; Mef2‐GAL4/+* were dissected in Schneider's *Drosophila* Medium (SDM). Dissected muscle preparations were maintained in SDM in a common imaging chamber and sequentially exposed to vehicle (SDM alone), 0.5 μM CCCP, and 100 μM CCCP by medium exchange, with 20 min of incubation in each condition before imaging. At each time point, 1–7 preparations were randomly selected for imaging.

### 2.4 MA‐5 Administration

Mitochonic acid‐5 (MA‐5) was kindly provided by the laboratory of Prof. Takaaki Abe. MA‐5 was dissolved in DMSO to prepare stock solutions and diluted to the indicated final concentrations immediately before use.

For acute ex vivo MA‐5 treatment in motor neurons (Figure 3B), adult flies aged 5 days after eclosion of the genotype *UAS‐QUEEN‐7μ/+; D42‐GAL4/+* were dissected in HL3.1 buffer. Thoracic ganglia were incubated in HL3.1 buffer containing 10 or 20 μM MA‐5 for 15 min at room temperature. Control samples were incubated in HL3.1 buffer containing the equivalent concentration of DMSO. Following treatment, samples were mounted in HL3.1 buffer and imaged on a Zeiss LSM980 confocal microscope under identical acquisition settings.

For acute ex vivo MA‐5 treatment in skeletal muscle (Figure 3A), adult flies of the genotype *UAS‐QUEEN‐7μ/+; Mef2‐GAL4/+* were dissected in SDM. Dissected muscle preparations were maintained in SDM in a common imaging chamber and sequentially exposed to vehicle, 100 nM MA‐5, and 10 μM MA‐5 by medium exchange. Each condition was incubated for 15 min. For the analysis shown in Figure 3A, the 15‐min endpoint of each condition was used.

For long‐term MA‐5 feeding in motor neurons (Figure 3C–E), MA‐5 was dissolved in DMSO at a stock concentration of approximately 100 mM and diluted in water before being thoroughly mixed into standard cornmeal food to yield a final MA‐5 concentration of 10 μM. The final DMSO concentration was 0.01% (v/v). Flies of the genotype *UAS‐QUEEN‐7μ/+; D42‐GAL4/+* were raised continuously on MA‐5‐supplemented food from the embryonic stage through adulthood. Control flies were raised on standard cornmeal food without MA‐5 supplementation. Flies were maintained at 25°C, and food was replaced every 3–5 days. Adult flies were collected at 5 days after eclosion, dissected in HL3.1 buffer, and thoracic ganglia were isolated, mounted, and imaged on a Zeiss LSM980 confocal microscope.

### 2.5 Antimycin A Treatment

For motor neuron experiments (Figure 1F–G), adult *Drosophila* aged 10 days post eclosion of the genotype *UAS‐QUEEN‐7μ/+; D42‐GAL4/+* were dissected in HL3.1 buffer. Thoracic ganglia were incubated in HL3.1 buffer containing 25 μM Antimycin A (Sigma‐Aldrich, A8674) for 3 min, followed by three 5‐min washes with HL3.1 buffer. Samples were mounted in HL3.1 buffer and imaged on a Zeiss LSM980 confocal microscope within 1 h.

For skeletal muscle experiments (Figure 1H–I), adult *Drosophila* aged 10 days post eclosion of the genotype *UAS‐QUEEN‐7μ/+; Mef2‐GAL4/+* were dissected in SDM. Dissected muscle preparations were equilibrated in SDM containing 100 μM Antimycin A (or SDM alone as vehicle control) for 15 min, then imaged in real time on a Zeiss LSM980 confocal microscope.

### 2.6 Immunostaining for Cleaved Dcp‐1

Adult thoracic ganglia from flies aged 10–20 days post eclosion were dissected in HL3.1 saline and incubated ex vivo in HL3.1 containing either vehicle; 0.5 μM, 10 μM, or 100 μM CCCP for 15 min; 100 μM CCCP for 2 h; or 5 μM staurosporine (STS) for 3 h at room temperature. STS was used as a positive control for apoptosis induction. Following treatment, tissues were fixed in 4% paraformaldehyde in PBS for 20 min and washed with PBST (0.3% Triton X‐100 in PBS). Samples were blocked in 5% normal goat serum in PBST for 1 h and incubated overnight at 4°C with rabbit anti‐cleaved Dcp‐1 antibody (Cell Signaling Technology, #9578, 1:500) and rat anti‐Elav antibody (DSHB, Rat‐Elav‐7E8A10, 1:500) to label neuronal nuclei. After washing, tissues were incubated with Alexa Fluor 488‐conjugated goat anti‐rabbit IgG (1:500) and Alexa Fluor 555‐conjugated goat anti‐rat IgG (1:500) for 2 h at room temperature. Samples were mounted in VECTASHIELD. Images were acquired using the same confocal microscope settings within each experiment.

### 2.7 *Drosophila* Embryo Collection

For embryo collection, 4‐ to 8‐day‐old crossed flies were transferred to grape juice‐agar plates supplemented with yeast paste. After 20 h, embryos deposited on the agar surface were collected and rinsed with distilled water. To remove the chorion membrane, embryos were treated with 50% bleach (sodium hypochlorite solution) for 30 s, followed by thorough rinsing with water. Dechorionated embryos were then gently dried and mounted on glass‐bottom dishes for imaging.

### 2.8 Confocal Imaging

QUEEN‐7μ is a ratiometric ATP biosensor whose 405ex/488ex excitation ratio reports intracellular ATP concentration (Yaginuma et al. 2014). Confocal images were acquired on a Zeiss LSM980 laser scanning microscope using a 20×/NA 0.8 or 40×/NA 1.3 oil immersion objective lens at room temperature (20°C–26°C). QUEEN‐7μ fluorescence was excited sequentially with 405 and 488 nm laser lines in frame‐sequential scan mode, and emission was collected through PMT (photomultiplier tube) detectors at a frame size of 1024 × 1024 pixels. Laser powers and detector gains were optimized to avoid saturation and kept identical across all samples within each experiment to ensure ratiometric comparability. For time‐course experiments (Figures 1E and 3A), samples were maintained at room temperature throughout image acquisition.

### 2.9 Quantification and Analysis

#### 2.9.1 Image Analysis

Confocal images were analyzed using Fiji software (ImageJ, NIH). For motor neuron preparations, individual cell bodies were used as regions of interest (ROIs); for skeletal muscle preparations, multiple areas of interest (AOIs) were defined per preparation. ROIs/AOIs were identified using the automated particle detection function in Fiji where feasible; in cases where automated detection was insufficient due to tissue morphology or image quality, ROIs were manually delineated to exclude aberrant signals. Mean fluorescence intensities at 405 and 488 nm excitation channels were measured for each ROI/AOI. The ratiometric QUEEN‐7μ signal was calculated as the ratio of the mean fluorescence intensity at 405 nm excitation to that at 488 nm excitation (405ex/488ex). For Figure 1G, background fluorescence measured from non‐fluorescent regions was subtracted prior to ratio calculation. For visualization, ratiometric images were generated by pixel‐wise division of the two channels and displayed using the Fire lookup table (LUT) in Fiji. For cleaved Dcp‐1 analysis, Dcp‐1‐positive cells within the thoracic ganglion were manually counted using Fiji. The number of positive cells per ganglion was used for statistical analysis.

#### 2.9.2 Normalization

**FIGURE 2:**
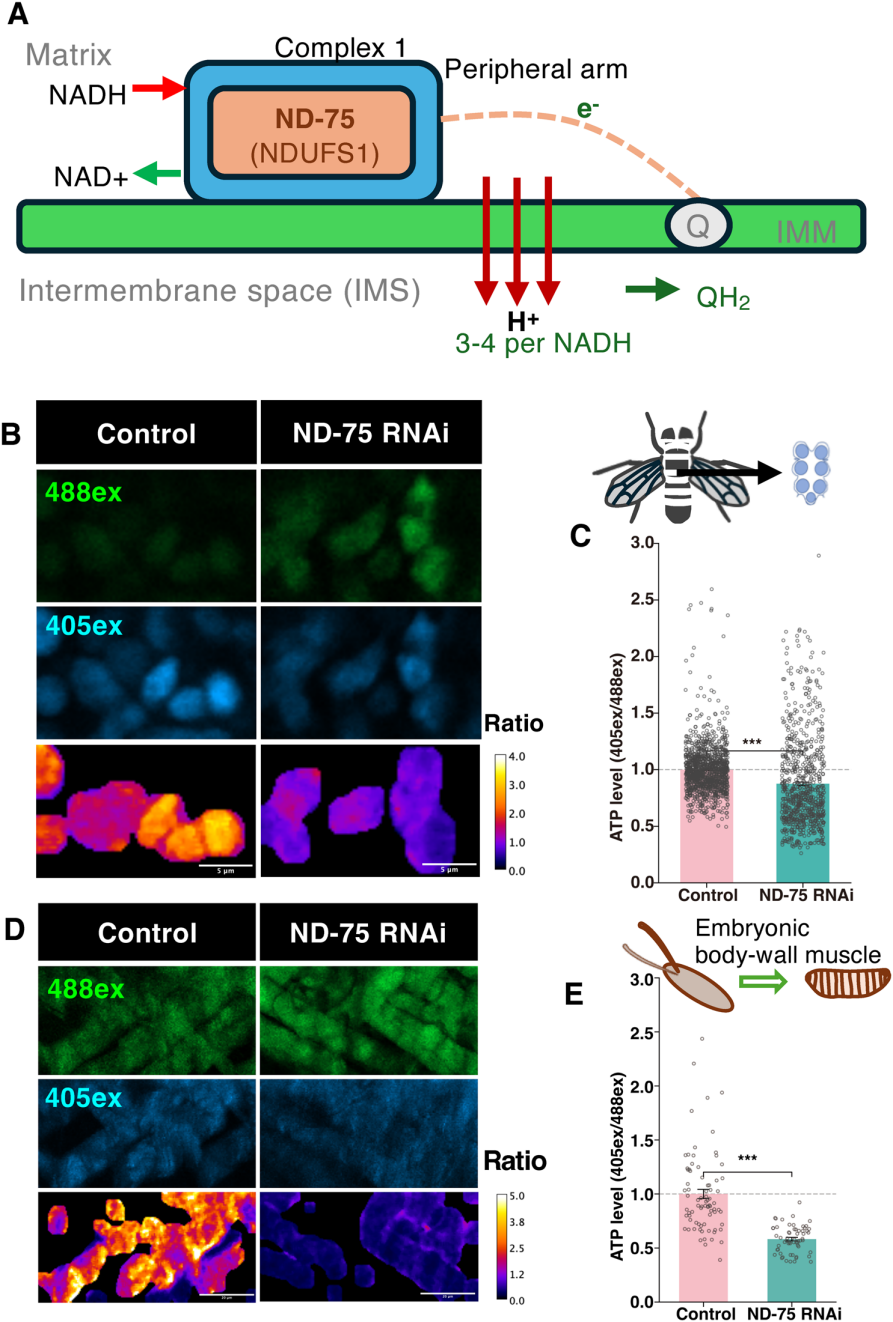
QUEEN‐7μ detects ATP reduction upon ND‐75 knockdown. (A) Schematic of mitochondrial Complex I and the location of ND‐75 (NDUFS1). ND‐75 is a core subunit of the peripheral arm of Complex I, where electrons derived from NADH are transferred to ubiquinone. This electron transfer contributes to proton translocation across the inner mitochondrial membrane, generating the proton motive force required for ATP synthesis. Q, ubiquinone; QH_2_, ubiquinol; IMS, intermembrane space; IMM, inner mitochondrial membrane. (B) Representative confocal images of thoracic ganglia from adult flies expressing QUEEN‐7μ in motor neurons, with or without ND‐75 RNAi expression. Images show 488ex excitation (top), 405ex excitation (middle), and pseudo‐colored 405ex/488ex ratio images (bottom, Fire LUT). (C) Quantification of the QUEEN‐7μ ratio in motor neurons, expressed as the 405ex/488ex fluorescence ratio normalized to control mean. Each data point represents an individual cell. Data are presented as mean ± SEM. ND‐75 RNAi expression significantly reduced the QUEEN‐7μ ratio compared with control (Welch's *t*‐test, two‐tailed; ***p < 0.001). *N* indicates the number of animals and *n* indicates the number of cells analyzed per group (control: *N* = 9, *n* = 1229; ND‐75 RNAi: *N* = 10, *n* = 777). (D) Representative confocal images of embryonic body‐wall muscle expressing QUEEN‐7μ, with or without ND‐75 RNAi expression. Images show 488ex excitation (top), 405ex excitation (middle), and pseudo‐colored 405ex/488ex ratio images (bottom, Fire LUT). (E) Quantification of the QUEEN‐7μ ratio in embryonic body‐wall muscle, expressed as the 405ex/488ex fluorescence ratio normalized to the control mean. Each data point represents an individual AOI. Data are presented as mean ± SEM. ND‐75 RNAi expression significantly reduced the QUEEN‐7μ ratio compared with control (Welch's *t*‐test, two‐tailed; ***p < 0.001). *N* indicates the number of animals and *n* indicates the number of AOIs analyzed per group (control: *N* = 6, *n* = 78; ND‐75 RNAi: *N* = 4, *n* = 60). Genotype: (B, C) *UAS‐QUEEN‐7μ/+; D42‐GAL4/+* (control), *UAS‐QUEEN‐7μ/+; UAS‐ND‐75 RNAi/D42‐GAL4* (ND‐75 RNAi) (D, E) *UAS‐QUEEN‐7μ/+; Mef2‐GAL4/+* (Control), *UAS‐QUEEN‐7μ/+; UAS‐ND‐75 RNAi/Mef2‐GAL4* (ND‐75 RNAi).

**FIGURE 3:**
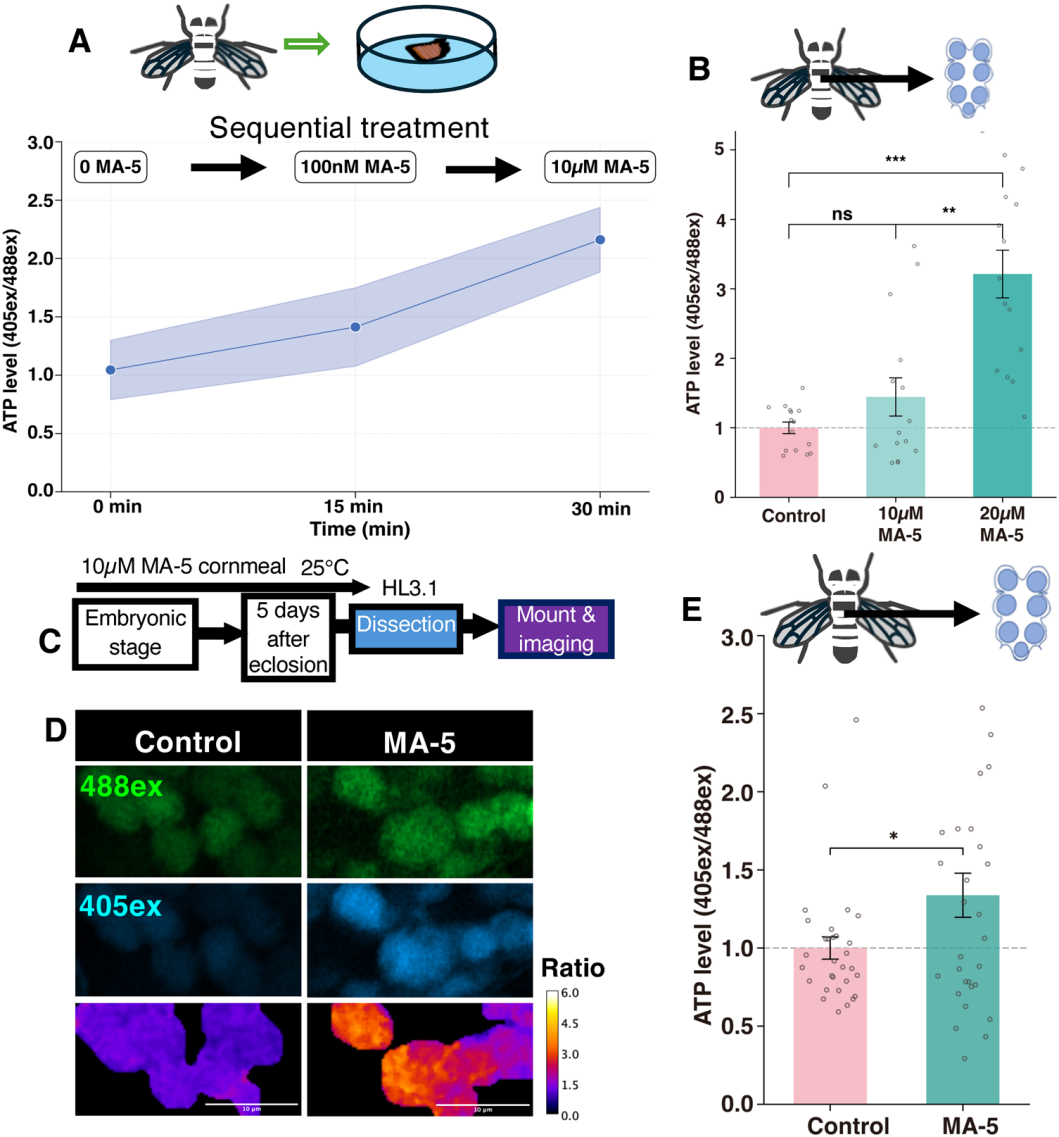
QUEEN‐7μ detects acute and chronic MA‐5‐induced increases in the ratio, consistent with elevated ATP levels. (A) Sequential exposure experiment in adult skeletal muscle expressing QUEEN‐7μ. Muscle preparations were maintained in a common imaging chamber and sequentially exposed to vehicle (0 MA‐5), 100 nM MA‐5, and 10 μM MA‐5 by buffer exchange (top schematic). Each condition was incubated for 15 min; values represent the 15‐min endpoint of each condition and are plotted at 0, 15, and 30 min along the cumulative timeline. The ratio is expressed as the 405ex/488ex fluorescence ratio normalized to the baseline value at 0 min. The QUEEN‐7μ ratio increased at both MA‐5 concentrations relative to vehicle baseline, with a larger increase at the higher concentration. Data were pooled from three independent experiments and are presented as mean ± SEM (shaded region). *N* = 3 independent experiments; *n* = 9 AOIs per condition (3 AOIs per experiment). (B) Quantification of the QUEEN‐7μ ratio in motor neurons following acute ex vivo MA‐5 treatment for 15 min, expressed as the 405ex/488ex fluorescence ratio normalized to the control mean. Each data point represents an individual cell. Data are presented as mean ± SEM. Acute MA‐5 treatment significantly increased the QUEEN‐7μ ratio in a dose‐dependent manner (Kruskal–Wallis test, *H* = 21.1, *p* = 2.6 × 10^−5^, followed by pairwise Mann–Whitney *U* tests with Bonferroni correction; control vs. 10 μM, *p* = 1.00, ns; control vs. 20 μM, *p* = 3.3 × 10^−5^, ***; 10 μM vs. 20 μM, *p* = 2.0 × 10^−3^, **). *N* indicates the number of animals and *n* indicates the number of cells analyzed per group (Control: *N* = 3, *n* = 15; 10 μM: *N* = 3, *n* = 15; 20 μM: N = 3, *n* = 15). (C) Schematic of the experimental workflow for MA‐5 feeding. Flies were raised on standard cornmeal food supplemented with 10 μM MA‐5 from the embryonic stage. Adult flies were collected 5 days after eclosion, dissected in HL3.1 buffer, and thoracic ganglia were mounted for imaging. (D) Representative confocal images of thoracic ganglia from adult flies expressing QUEEN‐7μ in motor neurons fed with standard food (control) or 10 μM MA‐5‐supplemented food. Images show 488ex excitation (top), 405ex excitation (middle), and pseudo‐colored 405ex/488ex ratio images (bottom, Fire LUT). (E) Quantification of the QUEEN‐7μ ratio in motor neurons after long‐term MA‐5 feeding, expressed as the 405ex/488ex fluorescence ratio normalized to the control mean. Each data point represents an individual cell. Data are presented as mean ± SEM. MA‐5 feeding significantly increased the QUEEN‐7μ ratio compared with control (Welch's *t*‐test, two‐tailed; *p < 0.05). *N* indicates the number of animals and *n* indicates the number of cells analyzed per group (Control: *N* = 6, *n* = 30; MA‐5: *N* = 6, *n* = 30). Genotype: (B, D, E) *UAS‐QUEEN‐7μ/+; D42‐GAL4/+*; (A) *UAS‐QUEEN‐7μ/+; Mef2‐GAL4/+*.

**FIGURE 4:**
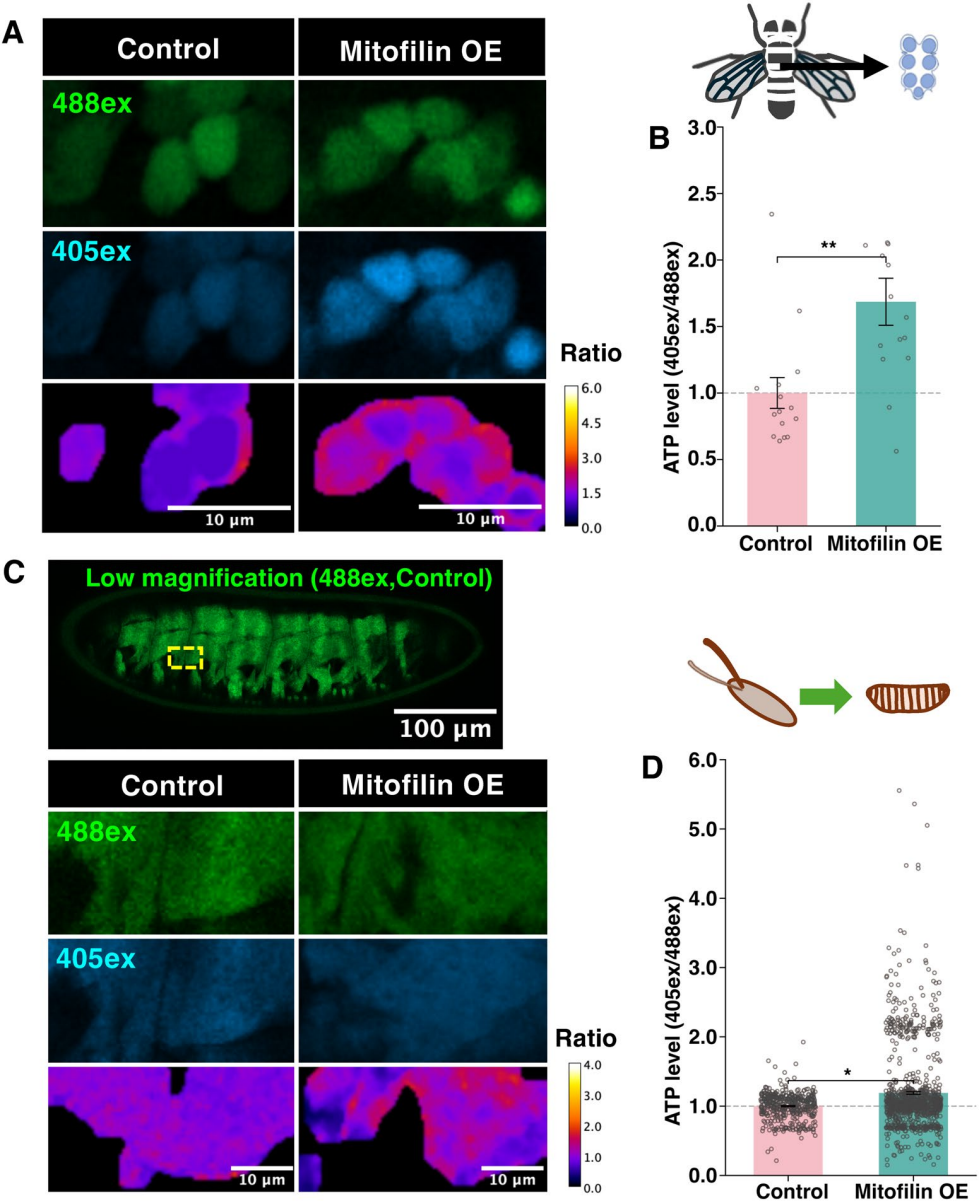
QUEEN‐7μ detects Mitofilin overexpression‐induced increases in the ratio, consistent with elevated ATP levels. (A) Representative confocal images of thoracic ganglia from adult flies expressing QUEEN‐7μ in motor neuron, with or without Mitofilin expression. Images show 488ex excitation (top), 405ex excitation (middle), and pseudo‐colored 405ex/488ex ratio images (bottom, Fire LUT). (B) Quantification of the QUEEN‐7μ ratio in motor neurons, expressed as the 405ex/488ex fluorescence ratio normalized to the control mean. Each data point represents an individual cell. Data are presented as mean ± SEM. Mitofilin overexpression significantly increased the QUEEN‐7μ ratio compared with control (Mann–Whitney *U* test, two‐sided; **p < 0.01). *N* indicates the number of animals and *n* indicates the number of cells analyzed per group (Control: *N* = 3, *n* = 15; MitofilinOE: *N* = 3, *n* = 15). (C) Low‐magnification overview of the embryonic body‐wall muscle preparation in the 488ex channel (control); the dashed box indicates the region used for high‐magnification analysis. Representative confocal images of embryonic body‐wall muscles expressing QUEEN‐7μ, with or without Mitofilin overexpression, are shown below. Images show 488ex excitation, 405ex excitation, and pseudo‐colored 405ex/488ex ratio images (Fire LUT). (D) Quantification of the QUEEN‐7μ ratio in embryonic body‐wall muscles, expressed as the 405ex/488ex fluorescence ratio normalized to the control mean. Each data point represents an individual AOI. Data are presented as mean ± SEM. Mitofilin overexpression significantly increased the QUEEN‐7μ ratio compared with control (Mann–Whitney *U* test, two‐sided; *p < 0.05). *N* indicates the number of animals and *n* indicates the number of AOIs analyzed per group (Control: *N* = 6, *n* = 452 AOIs; MitofilinOE: *N* = 12, *n* = 1039 AOIs). Genotype: (A, B) *UAS‐QUEEN‐7μ/+; D42‐GAL4/+* (Control), *UAS‐QUEEN‐7μ/UAS‐Mitofilin; D42‐GAL4/+* (MitofilinOE); (C, D) *UAS‐QUEEN‐7μ/+; Mef2‐GAL4/+* (Control), *UAS‐QUEEN‐7μ/UAS‐Mitofilin; Mef2‐GAL4/+* (MitofilinOE).

Two normalization schemes were used depending on the experimental design. Pool normalization, in which each ratio value was normalized to the mean ratio of the control group pooled across all experiments, was used for the data shown in Figures 1D,I, 2C,E, 3E, and 4B,D. Within‐experiment normalization, in which each ratio value was normalized to the mean ratio of the control group within the same experimental session, was used for the data shown in Figure 1G.

### 2.10 Climbing Assay (Negative Geotaxis)

Cohorts of 10–20 adult flies of each genotype (*D42‐GAL4/+*, *UAS‐Mitofilin/+*, and *UAS‐Mitofilin/+; D42‐GAL4/+*) were established within 24 h of eclosion and maintained at 25°C on standard cornmeal food, with food vials changed periodically. Climbing performance was assessed at days 5, 15, 25, and 35 post eclosion. At each timepoint, surviving flies from each cohort were gently transferred without anesthesia into clean glass test tubes and allowed to acclimate. Tubes were then tapped three times against a rubber‐cushioned benchtop to startle the flies to the bottom, and their upward locomotion was recorded by video for 20 s, with *t* = 0 defined as the moment of release from the final tap. Three sequential trials were performed per cohort with a 1‐min rest between trials. Videos were reviewed in iMovie (Apple), and the number of flies that failed to climb above a 5‐cm threshold (measured from the bottom of the tube) within 20 s was counted manually for each trial. The climbing pass rate per trial was calculated as [1 − (number of failed flies/number of surviving flies)], and the mean of the three trials was used as the climbing pass rate for that cohort at the corresponding timepoint. The denominator at each timepoint reflected the number of surviving flies; cohorts in which insufficient number of flies remained for reliable assessment were excluded from that timepoint. Statistical analysis was performed using two‐way ANOVA (Genotype × Age) followed by pairwise post hoc comparisons within each timepoint with Bonferroni correction; Mann–Whitney *U* tests were used as a nonparametric alternative. Each cohort therefore contributed a single climbing pass rate value per timepoint; in all figures, *n* refers to the number of independent cohorts assayed per group, with each cohort initially containing 10–20 flies.

### 2.11 Statistical Analysis

All statistical analyses and data visualization were performed using Python with the SciPy, NumPy, pandas, and Matplotlib libraries. The choice of statistical test was determined by the experimental design and data distribution. For the three‐group comparisons in Figures 1D and 3B, the Kruskal–Wallis test was followed by pairwise Mann–Whitney *U* tests with Bonferroni correction. For the two‐group comparisons in Figure 1G,I, the Mann–Whitney *U* test (two‐sided) was used. For the two‐group comparisons in Figures 2A,C,E, and 3E, Welch's *t*‐test (two‐tailed) was applied. For the two‐group comparisons in Figure 4B,D, the test was selected adaptively based on the Shapiro–Wilk normality test: Welch's *t*‐test (two‐tailed) was applied if both groups were normally distributed (*p* > 0.05); otherwise, the Mann–Whitney *U* test (two‐sided) was used. In both cases, the Mann–Whitney *U* test was selected because at least one group was not normally distributed. For the time‐course experiments in Figures 1E and 3A, descriptive analysis was performed without statistical comparisons. Statistical analysis of the Dcp‐1 experiment was performed using the Mann–Whitney *U* test (two‐sided), with each treatment group compared to the control. Significance levels are indicated as *p < 0.05, **p < 0.01, and ***p < 0.001. Data are presented as mean ± SEM, with individual data points shown where applicable.

## 3 Results

### 3.1 QUEEN‐7μ Detects ATP Depletion Induced by Mitochondrial Inhibitors in Drosophila Tissues

Previous studies have shown that QUEEN‐7μ quantitatively reports intracellular ATP levels in *Escherichia coli* ( *E. coli* ) (Yaginuma et al. 2014). To test whether QUEEN‐7μ can reliably monitor ATP dynamics in tissues of multicellular organisms, we generated transgenic flies carrying UAS‐QUEEN‐7μ. The insertion of the UAS‐QUEEN‐7μ transgene did not cause obvious developmental or morphological defects. As initial target tissues, we selected motor neurons and skeletal muscle because both are highly energy‐demanding cell types that depend strongly on mitochondrial ATP production while exhibiting distinct physiological and metabolic properties. Tissue‐specific expression of QUEEN‐7μ was confirmed in motor neurons (D42‐GAL4) and skeletal muscle (Mef2‐GAL4) by confocal imaging (Figure S1). To monitor ATP dynamics, adult tissues were dissected, treated with mitochondrial inhibitors, and imaged by ratiometric confocal microscopy (Figure 1A). These flies were viable and showed no obvious morphological abnormalities, suggesting that QUEEN‐7μ expression is well tolerated under these experimental conditions.

We first examined the response of QUEEN‐7μ to pharmacologically induced ATP depletion using mitochondrial inhibitors that have been widely used to perturb cellular ATP levels in previous biosensor studies (Imamura et al. 2009; Tsuyama et al. 2013). Motor neurons expressing QUEEN‐7μ were dissected from adult flies and treated with the mitochondrial uncoupler CCCP (Figure 1B). Confocal imaging revealed a clear reduction in the QUEEN‐7μ fluorescence ratio following CCCP treatment (Figure 1C). Quantification of the excitation ratio (405ex/488ex) showed a significant decrease in the QUEEN‐7μ ratio compared with untreated controls, with a greater reduction at the higher CCCP concentration, consistent with decreased ATP levels (Figure 1D).

We next tested whether QUEEN‐7μ also reports ATP changes in skeletal muscle. In adult skeletal muscle expressing QUEEN‐7μ, sequential exposure to CCCP—first at 0.5 μM and then at 100 μM over 40 min—resulted in a progressive decrease in the QUEEN‐7μ ratio (Figure 1E). This response suggests that QUEEN‐7μ can monitor inhibitor‐induced ATP depletion dynamically in muscle tissue.

To validate these findings using an independent perturbation of mitochondrial function, we treated tissues with Antimycin A, an inhibitor of mitochondrial Complex III (Figure 1B). Antimycin A similarly induced a robust decrease in the QUEEN‐7μ ratio in both motor neurons and skeletal muscle (Figure 1F–I). Together, these results show that QUEEN‐7μ ratio reliably decreases in response to pharmacological mitochondrial inhibition, consistent with ATP depletion across multiple *Drosophila* tissues.

Because severe mitochondrial inhibition can eventually induce cell death, we examined whether the decrease in the QUEEN‐7μ ratio observed under the strongest CCCP condition used for imaging was associated with apoptotic cell death. Apoptotic cells were detected by immunostaining for cleaved Dcp‐1, an established marker of apoptotic cell death in the *Drosophila* nervous system (Song et al. 1997; Kessissoglou et al. 2020). CCCP treatment for 15 min did not increase the number of Dcp‐1‐positive cells compared with control at any of the concentrations tested (0.5, 10, or 100 μM), including 100 μM, the highest concentration used for QUEEN‐7μ imaging, whereas prolonged CCCP treatment (100 μM, 2 h) showed a trend toward an increase in Dcp‐1‐positive cells that did not reach statistical significance. STS treatment robustly induced Dcp‐1 staining, confirming the sensitivity of the assay (Figure S2). These results indicate that, under the strongest CCCP condition used for imaging, the decrease in the QUEEN‐7μ ratio occurs before detectable apoptotic cell death.

### 3.2 Genetic Impairment of Mitochondrial Function Reduces the QUEEN‐7μ Ratio, Consistent With Decreased ATP Levels

To further validate whether QUEEN‐7μ detects physiologically relevant changes in ATP levels in vivo, we impaired mitochondrial ATP production using a genetic approach. We targeted ND‐75, which encodes a 75‐kDa iron–sulfur subunit of mitochondrial Complex I (NADH dehydrogenase), a component required for oxidative phosphorylation (Hegde et al. 2014; Granat et al. 2024) (Figure 2A). ND‐75 RNAi was expressed in motor neurons using the D42‐GAL4 driver in flies carrying the QUEEN‐7μ reporter. Confocal imaging revealed a clear reduction in the QUEEN‐7μ ratiometric signal in motor neurons expressing ND‐75 RNAi compared with controls (Figure 2B). Quantification of the excitation ratio (405ex/488ex), normalized to the control mean, confirmed a significant decrease in the QUEEN‐7μ ratio upon ND‐75 RNAi expression, to 87% of control, consistent with decreased ATP levels (Figure 2C; Welch's two‐tailed *t*‐test, *p* < 0.001; control: *N* = 9, *n* = 1229 cells; ND‐75 RNAi: *N* = 10, *n* = 777 cells).

We next examined whether similar effects were observed in a distinct tissue context. Expression of ND‐75 RNAi in embryonic body‐wall muscle also resulted in marked reduction in the QUEEN‐7μ ratio compared with controls (Figure 2D). Quantitative analysis confirmed a significant decrease in the QUEEN‐7μ ratio in muscles expressing ND‐75 RNAi, to 58% of control, consistent with decreased ATP levels (Figure 2E; Welch's two‐tailed *t*‐test, *p* < 0.001; control: *N* = 6, *n* = 78 AOIs; ND‐75 RNAi: *N* = 4, *n* = 60 AOIs).

Together, these results demonstrate that QUEEN‐7μ reports ATP reduction caused by genetic disruption of mitochondrial function across multiple *Drosophila* tissues.

### 3.3 QUEEN‐7μ Detects Acute and Chronic ATP Elevation Induced by MA‐5

After confirming that QUEEN‐7μ ratio decreases in response to ATP reduction, we next examined whether the sensor could also detect increases in ATP levels. MA‐5 is a small molecule originally identified by its ability to increase cellular ATP levels in cultured mammalian cells and has been reported to enhance mitochondrial ATP synthesis through interaction with Mitofilin/MIC60 and promotion of ATP synthase oligomerization (Suzuki et al. 2015; Matsuhashi et al. 2017).

To determine whether MA‐5 directly alters intracellular ATP level, independent of developmental or long‐term physiological effects, we first performed acute ex vivo treatment of adult skeletal muscle expressing QUEEN‐7μ. Muscle preparations were sequentially exposed to vehicle, 100 nM MA‐5, and 10 μM MA‐5 while continuously monitoring the QUEEN‐7μ ratio (Figure 3A). The QUEEN‐7μ ratio increased at both concentrations tested, with a larger increase at the higher concentration (Figure 3A). The ratio increased to approximately 140% of the control level after treatment with 100 nM MA‐5 and to approximately 215% after treatment with 10 μM MA‐5, demonstrating a rapid and graded response consistent with elevated ATP levels. These findings indicate that QUEEN‐7μ detects acute ATP‐associated changes induced by MA‐5 before substantial secondary physiological adaptation can occur.

We performed an acute MA‐5 treatment in motor neurons (ex vivo, 15 min), analogous to the acute muscle experiment (Figure 3B). Acute MA‐5 treatment significantly increased the neuronal QUEEN‐7μ ratio in a dose‐dependent manner, reaching approximately 3.2‐fold of control at 20 μM (Figure 3B; Kruskal–Wallis test, *H* = 21.1, *p* = 2.6 × 10^−5^, followed by pairwise Mann–Whitney *U* tests with Bonferroni correction; control vs. 10 μM, *p* = 1.00, ns; control vs. 20 μM, *p* = 3.3 × 10^−5^; 10 μM vs. 20 μM, *p* = 2.0 × 10^−3^; *N* = 3 animals, *n* = 15 cells per group), indicating a direct, acute effect on the neuronal QUEEN‐7μ ratio, consistent with elevated ATP levels and with the acute muscle result, independent of the potential confounds of long‐term incubation.

We next examined whether sustained MA‐5 administration similarly increased ATP levels in vivo. Flies expressing QUEEN‐7μ in motor neurons were raised on standard cornmeal food supplemented with 10 μM MA‐5 from the embryonic stage to adulthood to ensure sustained exposure to the compound (Figure 3C). No obvious morphological or developmental abnormalities were observed in MA‐5‐treated flies compared with control animals. Adult flies were collected 5 days after eclosion, and thoracic ganglia were imaged by confocal microscopy under identical imaging conditions. Compared with control flies raised on standard food, MA‐5‐treated flies exhibited a clear increase in the QUEEN‐7μ ratiometric signal (Figure 3D). Quantification of the excitation ratio (405ex/488ex), normalized to the control mean, revealed a significant elevation in the QUEEN‐7μ ratio in motor neurons after MA‐5 treatment, consistent with increased ATP levels (Figure 3E; Welch's two‐tailed *t*‐test, *p* < 0.05; control: *N* = 6, *n* = 30 cells; MA‐5: *N* = 6, *n* = 30 cells).

Together, these results demonstrate that the QUEEN‐7μ detects ATP‐associated increases induced by both acute pharmacological stimulation and prolonged in vivo administration of MA‐5, indicating that the reporter monitors ATP elevation across distinct experimental paradigms.

### 3.4 Mitofilin Overexpression Increases the QUEEN‐7μ Ratio and Improves Motor Function in Drosophila

Having shown that MA‐5 increases the QUEEN‐7μ ratio, consistent with elevated ATP levels, we next asked whether genetic manipulation of its reported target could produce a similar response. MA‐5 has been reported to interact with Mitofilin, also known as MIC60, a core component of the MICOS (mitochondrial contact site and cristae organizing system) complex that regulates mitochondrial cristae organization and supports efficient oxidative phosphorylation (Pfanner et al. 2014; Suzuki et al. 2016; Matsuhashi et al. 2017; Kozjak‐Pavlovic 2017). Because Mitofilin is linked to mitochondrial inner membrane architecture and ATP synthase organization, we tested whether Mitofilin overexpression was sufficient to increase the QUEEN‐7μ ratio in *Drosophila* tissues.

Mitofilin was overexpressed in motor neurons together with QUEEN‐7μ using the D42‐GAL4 driver. Confocal imaging revealed an increase in the QUEEN‐7μ ratiometric signal compared with controls (Figure 4A). Quantification of the excitation ratio (405ex/488ex), normalized to the control mean, confirmed a significant elevation in the QUEEN‐7μ ratio, consistent with increased ATP levels (Figure 4B; Mann–Whitney *U* test, two‐sided, *p* < 0.01; control: *N* = 3, *n* = 15 cells; MitofilinOE: *N* = 3, *n* = 15 cells).

We next tested whether a similar response could be detected in muscle. In embryonic body‐wall muscle expressing QUEEN‐7μ and Mitofilin, Mitofilin overexpression also resulted in a clear increase in the QUEEN‐7μ ratio compared with controls (Figure 4C). Quantitative analysis confirmed a significant increase in the QUEEN‐7μ ratio in muscle tissue, consistent with increased ATP levels (Figure 4D; Mann–Whitney *U* test, two‐sided, *p* < 0.05; control: *N* = 6, *n* = 452 AOIs; MitofilinOE: *N* = 12, *n* = 1039 AOIs).

Because motor function depends on the coordinated activity of motor neurons and skeletal muscles, we next examined whether Mitofilin overexpression also affects organismal motor performance. Motor neuron–specific Mitofilin overexpression attenuated age‐related decline in climbing performance in adult flies (Figure S3). These results suggest that Mitofilin‐mediated increases in the QUEEN‐7μ ratio are associated with improved motor function in vivo.

Together, these findings show that QUEEN‐7μ detects ATP elevation induced by genetic modulation of Mitofilin/MIC60 and support the utility of this reporter for linking mitochondrial bioenergetics to tissue and organismal function.

## 4 Discussion

In this study, we established a transgenic *Drosophila* system expressing the ratiometric ATP biosensor QUEEN‐7μ and demonstrated its applicability for monitoring cellular energy status in vivo. The principal advance of this work is the extension of QUEEN to a genetically tractable multicellular organism, enabling tissue‐specific and quantitative analysis of ATP dynamics in vivo. A key feature of this system is its ability to detect bidirectional changes in the QUEEN‐7μ ratio, consistent with changes in ATP levels. Pharmacological and genetic perturbations that impair mitochondrial function decreased the ratio, whereas MA‐5 treatment and Mitofilin overexpression increased it. Because intracellular ATP reflects the balance between production and consumption, this bidirectional responsiveness is essential for interpreting metabolic states and supports the use of QUEEN‐7μ as a robust readout of mitochondrial bioenergetics in vivo.

The ratiometric design of QUEEN provided practical advantages for in vivo imaging in *Drosophila* tissues. By reducing the impact of sensor expression variability, focal drift, tissue thickness, and batch‐to‐batch variation, the excitation‐ratio readout improved quantitative reliability in intact tissues. These features were particularly important for detecting relatively modest ATP‐associated changes across individuals and tissue regions. FRET‐based ATP sensors such as ATeam have been successfully applied in cultured cells and select *Drosophila* tissues (Imamura et al. 2009; Tsuyama et al. 2013). QUEEN provides a complementary single‐fluorophore ratiometric approach that is well suited for cross‐sample comparisons in genetically defined tissues.

Compared with ATP reduction, experimentally induced ATP elevation is generally more difficult to demonstrate in vivo, likely because intracellular ATP concentrations are maintained within a relatively narrow physiological range by homeostatic metabolic regulation (Atkinson 1968; Chapman et al. 1971). In this context, the observation that both MA‐5 treatment and Mitofilin overexpression increased the QUEEN‐7μ ratio is notable and consistent with previous evidence that reinforcement of MIC60‐dependent mitochondrial organization can improve mitochondrial bioenergetic capacity (Tsai et al. 2018). The ability of QUEEN‐7μ to detect such sustained ATP‐associated increases in vivo expands the utility of ATP imaging approaches.

Our results also support a functional link between mitochondrial inner membrane organization and ATP production. MA‐5 has been reported to interact with Mitofilin/MIC60 and facilitate ATP synthase oligomerization, thereby promoting ATP production (Suzuki et al. 2015; Suzuki et al. 2016; Matsuhashi et al. 2017). Mitofilin/MIC60 is a core component of the MICOS complex, which organizes cristae junctions and maintains cristae architecture, processes closely linked to oxidative phosphorylation efficiency (Pfanner et al. 2014; Kozjak‐Pavlovic 2017). Consistent with this view, MIC60 is required for proper crista structure and mitochondrial function in *Drosophila* (Tsai et al. 2017), and MIC60 overexpression has been reported to restore ATP levels in PINK1‐null mutants (Tsai et al. 2018). Our observation that Mitofilin overexpression increases the QUEEN‐7μ ratio in both motor neurons and muscle further suggests that Mitofilin‐dependent regulation of mitochondrial architecture may influence ATP production in vivo.

We also observed tissue‐dependent differences in the variability of the QUEEN‐7μ ratio, with skeletal muscle exhibiting greater heterogeneity than motor neurons. This likely reflects intrinsic differences in metabolic demand and flexibility between these tissues (Harris et al. 2012; Rangaraju et al. 2014) and highlights the potential of this system to capture physiologically relevant metabolic diversity across cell types.

Several limitations of the present study should be acknowledged. Although the present data support concentration‐dependent responses under the tested conditions, a finer dose–response series will be required to define the detection range and limits of the sensor. At high concentrations of mitochondrial inhibitors, ATP depletion may be accompanied by loss of cell viability, which should be considered when interpreting lower bound sensor responses. In addition, although the ratiometric design improves robustness against expression variability, absolute comparison of ATP concentrations across tissues may require calibration under defined conditions, and optical factors such as photobleaching may influence signal stability during prolonged imaging.

Overall, the QUEEN reporter system provides a robust and versatile platform for in vivo analysis of ATP dynamics in *Drosophila*. By enabling quantitative measurement of cellular energy status in genetically defined cell populations, this system can be integrated with genetic and pharmacological approaches and will facilitate future studies of mitochondrial function and energy metabolism in development, differentiation, physiology, and disease models.

## Author Contributions

Y.L. performed all experiments and analyzed the data. S.S. generated the QUEEN transgenic lines. N.K., H.U., T.A., and T.T. assisted with the QUEEN experiments. E.K. conceived and supervised the study and wrote the manuscript.

## Funding

This work was supported by Japan Society for the Promotion of Science (21H05255, 24H00564, JP24687027, 16H04800, 24K22012, 26H00967), Japan Agency for Medical Research and Development (25zf0127001h1105), Japan Science and Technology Agency (JPMJCR1852, JPMJFR233A), Takeda Science Foundation, Japan Foundation for Applied Enzymology, Uehara Memorial Foundation, Mitsubishi Foundation, Naito Foundation.

## Conflicts of Interest

The authors declare no conflicts of interest.

## Supporting information


**Figure S1:** Tissue‐specific expression of QUEEN‐7μ in *Drosophila*.
**Figure S2:** Short‐term CCCP treatment used for ATP imaging does not induce detectable apoptosis.
**Figure S3:** Motor neuron–specific Mitofilin overexpression attenuates age‐related decline in climbing performance.

## Data Availability

The data that support the findings of this study are available from the corresponding author upon reasonable request.
