# Supplementary material for: Quantitative In Vivo Imaging of ATP Dynamics in Drosophila Using the Ratiometric Biosensor QUEEN
Source: Dev Growth Differ. 2026 Aug 3;68(6):e70065. doi: 10.1111/dgd.70065 (PMC13433815; doi:10.1111/dgd.70065)
Supplement: Supplementary file 1 [file DGD-68-0-s001.pdf]

Supplementary Figure 1

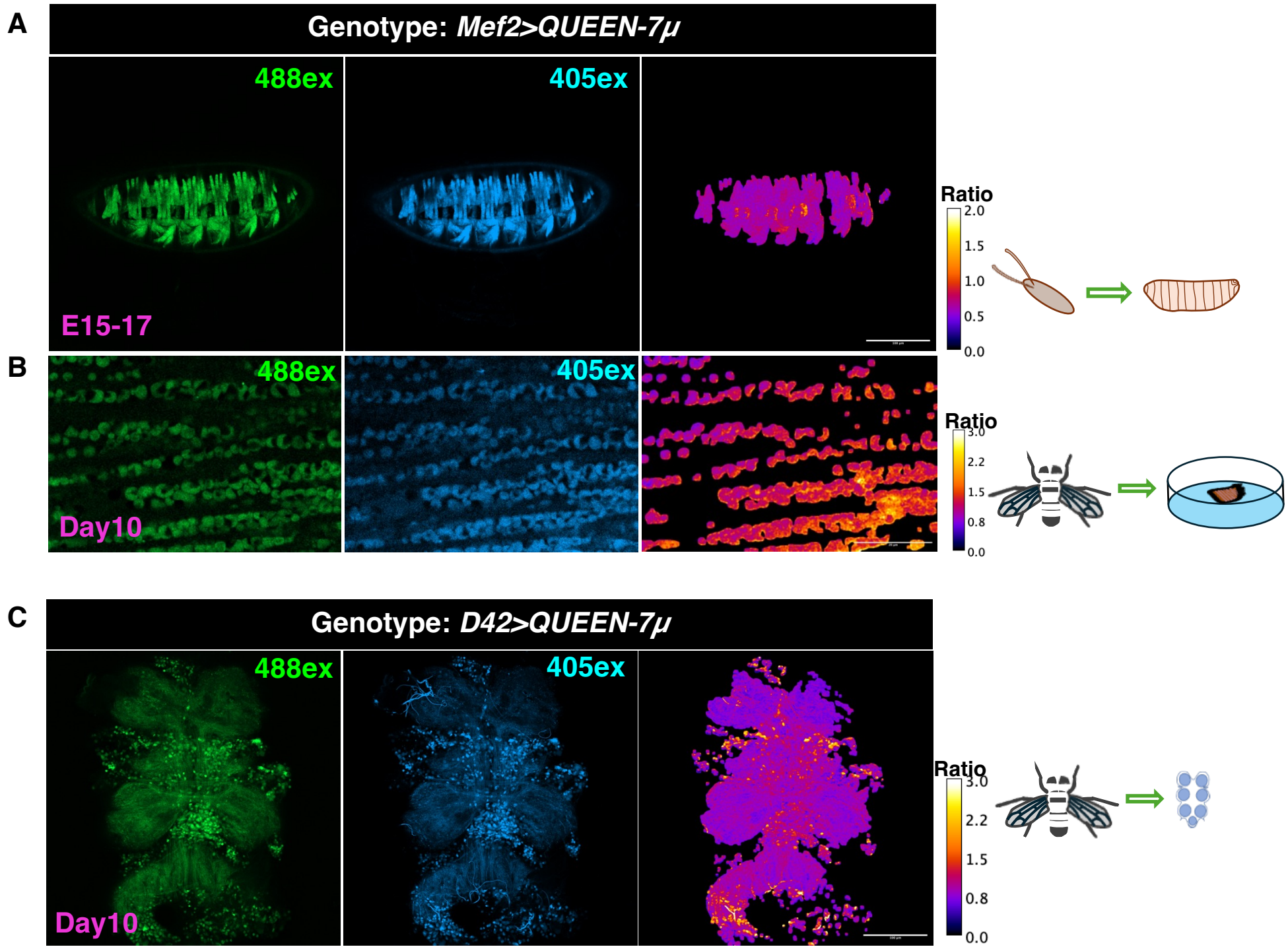

Supplementary Figure 1. Tissue-specific expression of QUEEN-7μ in *Drosophila*.

Representative confocal images showing QUEEN-7μ expression driven by tissue-specific GAL4 drivers at different developmental stages. (A) Mef2-GAL4-driven QUEEN-7μ expression in embryonic body-wall muscle at stage E15-17. (B) Mef2-GAL4-driven QUEEN-7μ expression in adult skeletal muscle at day 10 after eclosion. (C) D42-GAL4-driven QUEEN-7μ expression in adult thoracic ganglia at day 10 after eclosion. For each preparation, 488ex excitation (left), 405ex excitation (middle), and pseudocolored 405ex/488ex ratio images (right, Fire LUT) are shown. Scale bars: 100 μm (A, C), 20 μm (B).

Genotype: (A, B) *UAS-QUEEN-7μ/+; Mef2-GAL4/+* (Mef2>QUEEN-7μ), (C) *UAS-QUEEN-7μ/+; D42-GAL4/+* (D42>QUEEN-7μ)

Supplementary Figure 2

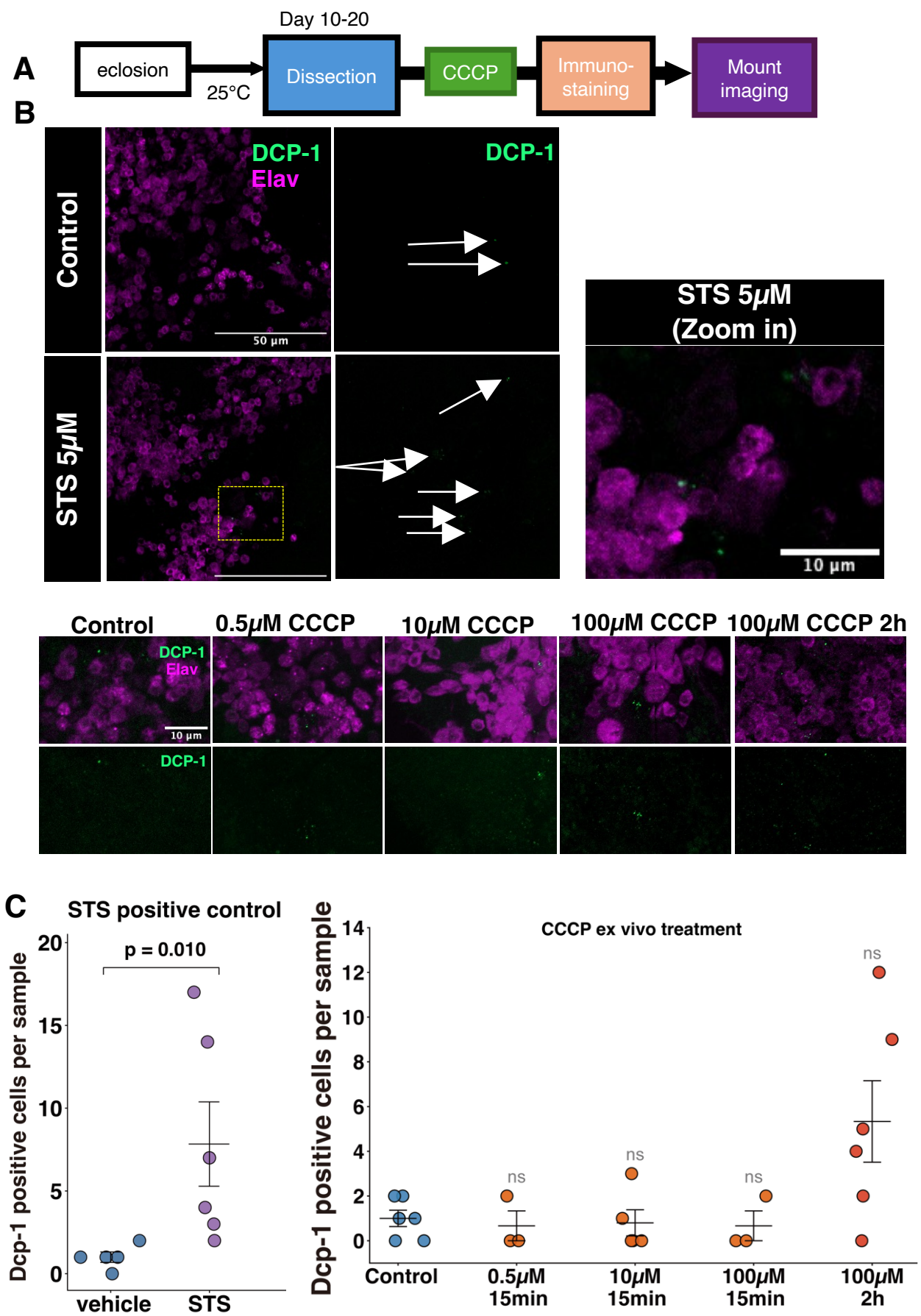

Supplementary Figure 2. Short-term CCCP treatment used for ATP imaging does not induce detectable apoptosis.

(A) Experimental design. Adult flies were aged for 10–20 days at 25 °C after eclosion. Thoracic ganglia were then dissected and incubated ex vivo with vehicle, 0.5, 10, or 100 µM CCCP for 15 min (100 µM corresponding to the condition used for ATP imaging), or 100 µM CCCP for 2 h. As an independent positive control, ganglia were treated with 5 µM staurosporine (STS) for 3 h. Ganglia were then immunostained for cleaved Dcp-1 and mounted for confocal imaging.

(B) Representative confocal images of adult thoracic ganglia immunostained for cleaved Dcp-1, an established marker of apoptotic cells in the *Drosophila* nervous system. Neuronal nuclei were labeled with anti-Elav. Scale bar, 50 µm (STS and control overview), 10 µm (magnified inset and CCCP-treatment panels).

(C) Quantification of cleaved Dcp-1-positive cells per thoracic ganglion. No significant increase in apoptotic cells was observed after 15 min CCCP treatment compared with the control; at any of the concentrations tested (0.5, 10, or 100 µM) compared with control; prolonged CCCP treatment (100 µM, 2 h) showed a trend toward an increase that did not reach significance, whereas STS treatment significantly increased the number of cleaved Dcp-1-positive cells. Data are presented as mean ± SEM. Statistical analysis was performed using Mann-Whitney U test (two-sided). N indicates the number of thoracic ganglia analyzed per group (Control: N=6; 0.5 µM CCCP: N=3; 10 µM CCCP: N=4; 100 µM CCCP 15 min: N=3; 100 µM CCCP 2 h: N=6; vehicle: N=5; STS: N=6).

Supplementary Figure 3

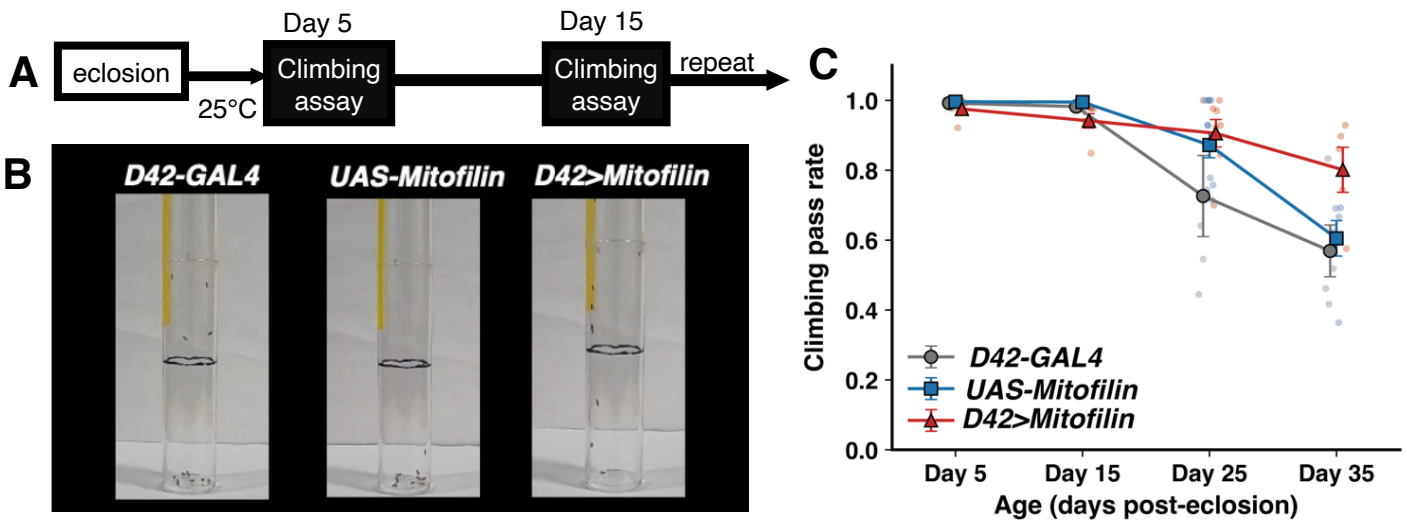

Supplementary Figure 3. Motor neuron-specific Mitofilin overexpression attenuates age-related decline in climbing performance.

(A) Schematic of the climbing assay workflow. Adult flies were collected at eclosion and maintained on standard cornmeal food at 25°C. Climbing performance was assessed at 5, 15, 25, and 35 days after eclosion.

(B) Representative images from climbing assay videos at the 20-s endpoint for each genotype. The yellow line indicates the 5-cm threshold used for scoring. Flies reaching or exceeding this height were scored as successful.

(C) Quantification of climbing performance across age. The climbing pass rate was calculated as the percentage of flies that crossed the 5-cm threshold within 20 s. Large symbols indicate mean ± SEM, and small transparent symbols represent individual cohort values (mean of three trials per cohort). Two-way ANOVA revealed significant main effects of genotype ($F(2, 63) = 4.09$, $p = 0.021$) and age ($F(3, 63) = 31.39$, $p < 0.001$), as well as a significant genotype × age interaction ($F(6, 63) = 2.52$, $p = 0.030$), indicating that Mitofilin overexpression modifies the age-dependent trajectory of motor performance. Pairwise post-hoc comparisons within individual time points did not reach statistical significance after Bonferroni correction. Each cohort contained 10–20 flies, and the pass rate was calculated using the number of surviving flies at each time point. n indicates the number of independent cohorts analyzed per genotype.

Genotype: (B, C) *D42-GAL4/+* (as driver control), *UAS-Mitofilin/+* (as transgene control), *UAS-Mitofilin/+; D42-GAL4/+* (D42>Mitofilin).

Day 5: *D42-GAL4/+* (n = 3), *UAS-Mitofilin/+* (n = 10), *UAS-Mitofilin/+; D42-GAL4/+* (n = 6).

Day 15: *D42-GAL4/+* (n = 3), *UAS-Mitofilin/+* (n = 9), *UAS-Mitofilin/+; D42-GAL4/+* (n = 6).

Day 25: *D42-GAL4/+* (n = 5), *UAS-Mitofilin/+* (n = 10), *UAS-Mitofilin/+; D42-GAL4/+* (n = 7).

Day 35: *D42-GAL4/+* (n = 5), *UAS-Mitofilin/+* (n = 6), *UAS-Mitofilin/+; D42-GAL4/+* (n = 5).
